# A Comparison of the Prevalence of the Parasites of the Digestive Tract in Goats from Organic and Conventional Farms

**DOI:** 10.3390/ani11092581

**Published:** 2021-09-02

**Authors:** Bogumiła Pilarczyk, Agnieszka Tomza-Marciniak, Renata Pilarczyk, Elżbieta Bombik, Beata Seremak, Jan Udała, Nikola Sadowska

**Affiliations:** 1 Department of Animal Reproduction Biotechnology and Environmental Hygiene, Faculty of Biotechnology and Animal Husbandry, West Pomeranian University of Technology, Szczecin, 71-270 Szczecin, Poland; bogumila.pilarczyk@zut.edu.pl (B.P.); beata.seremak@zut.edu.pl (B.S.); jan.udala@zut.edu.pl (J.U.); nikola.sadowska@zut.edu.pl (N.S.); 2 Laboratory of Biostatistics, Faculty of Biotechnology and Animal Husbandry, West Pomeranian University of Technology, Szczecin, 71-270 Szczecin, Poland; renata.pilarczyk@zut.edu.pl; 3 Faculty of Agrobioengineering and Animal Husbandry, Siedlce University of Natural Sciences and Humanities, 08-110 Siedlce, Poland; elzbieta.bombik@uph.edu.pl

**Keywords:** parasites, goats, conventional farming, organic farming, gastrointestinal nematodes, *Eimeria* spp., *Moniezia expansa*, *Fasciola hepatica*

## Abstract

**Simple Summary:**

The presence of gastrointestinal parasites such as coccidia (protozoa), gastrointestinal nematodes, flukes, and tapeworms are a considerable problem in goat keeping. Parasitic infections cause a deterioration of animal health, delay in growth rate, weight loss, reduced milk production, and miscarriages. The aim of the study was to compare the prevalence and intensity of parasitic infections observed in the digestive tracts of goats kept on organic and conventional farms. Our findings indicate that conventional goat herds demonstrate a similar prevalence of parasitic diseases as organic herds. Nevertheless, the prophylactic programs used to combat parasitic infections in both types of farms appear ineffective and require improvement. There is a need for goat herds to be covered by ongoing parasitological monitoring, including parasitological testing before and after the pasture season, to detect carriers and shedders of parasite eggs, oocysts, and cysts. It is also recommended that keepers employ rotational or intensive rotational grazing methods and take care to ensure the hygiene of animal quarters and livestock rooms. Furthermore, accurate diagnosis of parasitic infections, as well as effective monitoring and prophylaxis, are essential for keeping goat herds free from parasitic infections.

**Abstract:**

The aim of the study was to compare the prevalence and intensity of gastrointestinal parasitic infections in goats kept on organic (*n* = 76) and conventional farms (*n* = 82). In general, a higher prevalence of some gastrointestinal parasitic infections was found in the conventional farms compared to the organic farms: the mean prevalence of *Eimeria* spp. was 85.4% in conventional farms and 77.6% in organic farms, that of *Fasciola hepatica* was 6.10% in conventional farms and 2.63% in organic farms, and that of *Moniezia expansa* was 31.7% and 17.1%, in conventional and organic farms, respectively. Both farm types demonstrated a similar mean prevalence of nematodes (80.3 vs. 84.2%). Conventional farms demonstrated a significantly higher intensity of infection with *E. arloingi*, *Haemonchus* spp., *Nematodirus* spp. and *Moniezia expansa* compared to organic farms. They also demonstrated a higher intensity of infection with *Eimeria* spp. than organic farms. The prophylactic programs used to combat parasitic infections in both types of farms appear ineffective and require improvement. There is a need for goat herds to be covered by ongoing parasitological monitoring. It is also recommended that keepers employ rotational or intensive rotational grazing methods and take care to ensure the hygiene of animal quarters and livestock rooms.

## 1. Introduction

Recent years have seen a growth in the number of organic farms, particularly since some countries have favourable conditions for their development. Many provide various economic incentives for organic production, such as financial support from various national and EU programs, growing demand and ease of marketability, lower production costs and higher product prices. Organic methods are also widely regarded as being more environmentally friendly and better for animal welfare. Food produced based on natural methods is seen as healthy and is appreciated by consumers [1,2].

However, animals kept under organic conditions are more exposed to the risk of contracting infectious diseases, including parasitoses, with one reason being the limited use of antiparasitic drugs compared to conventional farms. In organic animal husbandry, disease prevention or control relies mainly on non-chemical methods. Although synthetic antiparasitic drugs can be used in organic farms (under the conditions regulated by the legal EU and national regulations [3]), parasites are usually controlled by authorised drugs based on phytotherapy [4].

Data on the parasitosis of animals kept in organic farms is scarce, and those that are available show a high proportion of infected animals, often by several parasites [5,6]. The most common parasitoses noted in goats from organic farms are those of the gastrointestinal nematodes and *Eimeria* spp., while *Moniezia* spp. and *Fasciola hepatica* are recorded less frequently, and they are also major preoccupations for organic farmers [4,7].

These groups of parasites include numerous pathogenic species capable of causing disease, e.g., *Eimeria arloingi*, *Haemonchus contortus* [8,9] or *Trichostrongylus colubriformis*, as well as less pathogenic species, including *E. hirci*, *E. punctata*, *Nematodirus* spp. [10]. The presence of these parasites is a considerable problem in goat keeping because they cause a deterioration of animal health, delay in growth rate, weight loss and miscarriages, as well as increased costs associated with production and veterinary treatment, and even death [11,12,13,14]. In addition, there are costs related to the decrease in animal productivity (weaker weight gain in meat breeds, a decrease in milk production in dairy breeds). Hoste and Chartier [15] noted that subclinical infection caused by *H. contortus* and *T. colubriformis* induced a decrease in body condition score and persistent decrease in milk yield, ranging from 2.5–10% in goats with the lowest performance and 12–25% in goats with the highest milk production. A significant decrease in the fat content of milk was also observed. In turn, Kyriánová et al. [5] reported that high intensity of strongylid infection contributed to a significant decrease (*p* < 0.01) in milk protein. These changes in the composition of milk may be reflected in its price and limit the technological suitability of goat milk [16]. According to Charlier et al. [17], the annual estimated costs of helminth infections in dairy goats in Europe was 67–107 million €.

Unfortunately, parasitic diseases are often underestimated by farmers, and deworming is not always preceded by a faecal examination for parasites. In addition, vets often assume a goat to respond the same way as a sheep or a cow and use incorrect drug dosing. Goats have a more rapid metabolism of anthelmintics than sheep. Therefore, applying the same doses to goats as to sheep may promote more rapid selection for resistance in parasites. However, care must be taken to avoid poisoning when increasing the dose rate of anthelmintics not registered for goats [18,19,20,21]. Therefore, as the main site of infection for goats is the pasture, an important element in the protection of small ruminants against parasitic diseases in organic farms is correct management of grazing. Maintaining so-called safe pastures, i.e., those free from invasive parasites, can reduce or eliminate parasitoses in the herd [4].

The aim of the study was to compare the prevalence and intensity of parasitic infections observed in the digestive tracts of goats kept on organic and conventional farms.

## 2. Materials and Methods

The faeces from goats from 2 organic farms and 4 conventional farms were subjected to coproscopy examination in September 2019. In total, 76 goats were studied from the organic farms and 82 goats from conventional farms, located in the West Pomerania region of NW Poland. Faecal samples (≈10 g) were collected from the rectum of animals and placed in labelled plastic bags. The samples were transported and refrigerated at 4 °C. Laboratory analyses were performed within 48 h.

Both the goats on organic and conventional farms were kept on deep bedding. In the conventional farms, deworming was carried out twice a year: once in spring before pasture, and again in autumn after pasture (levamisole hydrochloride, 8%). In the organic farms, deworming was carried out when needed (after examination) using drugs with the same active substance as in conventional farms. The information on farms obtained from the herd owners is given in Table 1.

Infection with gastrointestinal nematodes, *Eimeria* spp. and *Moniezia expansa* tapeworms was determined on the basis of coproscopic examinations using the Willis–Schlaaf flotation method [21]. The intensity of infection was determined quantitatively by the McMaster method [21]. Larval cultures were obtained from the isolated eggs, and these were used to identify the gastrointestinal nematodes to genus level [22,23]. Liver fluke eggs were detected by decantation [24].

The species composition of coccidia in the studied goats was determined based on oocyst morphology (shape, colour, form index), the time of sporulation, as well as the presence or absence of a micropyle and its caps, residual bodies, polar bodies and Stied bodies. The oocysts were cultured in a moist chamber at a temperature of 24–26 °C, with a 2.5% aqueous solution of potassium dichromate (K_2_Cr_2_O_7_). Species composition was established based on keys provided by Chartier and Paraud [9].

The results were statistically analysed with Statistica 13.3 (TIBCO Software Inc., Palo Alto, CA, USA). The χ^2^ test was used to compare the prevalence between particular species of parasites on organic and conventional farms, while the intensity of infection was examined using the Mann–Whitney U-test. Differences were determined to be statistically significant at *p* < 0.05. The confidence interval of the proportion was calculated by the modified Wald method, as recommended by Agresti and Coull [25].

## 3. Results

In general, the mean prevalence of gastrointestinal tract parasitic infections was comparable (*p* = 0.658) between conventional farms and organic farms (Table 2).

The mean prevalence of *Eimeria* spp. was 85.4% in conventional farms and 77.6% in organic farms (Table 3). Conventional farms demonstrated a significantly higher prevalence of infection with *E. arloingi* (χ^2^ = 5.16; *p* = 0.023), compared to organic farms. For other species, the difference was not statistically significant. Conventional farms also demonstrated a significantly higher intensity of infection (Z = −2.15; *p* = 0.031) compared to organic farms. However, statistically significant differences were noted only for 1 species—*E. jolchijevi* (Z = −2.88; *p* = 0.004).

Many animals were infected with several coccidia species. Single and multi-species *Eimeria* infections in goats on organic farms are presented in Table 4. Mixed infections were found in 73.7% of studied goats. The most common mixed infections were those comprising three (19.77%) and four (27.68%) species of *Eimeria*. Among the three-species co-infections, the presence of *E. caprina* (*n* = 8) and *E. chrisienseni* (*n* = 8) was most frequently observed. In the case of four-species co-infections, *E. alijevi* was most common (*n* = 11). Single-species infections were found in 13 goats, and the most widespread was *E. arloingi* (*n* = 5).

Single and multi-species infections in goats on conventional farms are presented in Table 5. Mixed infections were found in 81.7% of studied goats. The most common mixed infections were those comprising three (29.27%) and four (29.76%) species of *Eimeria*. Among the three-species co-infections, the presence of *E. arloingi* was most frequently observed (*n* = 18). In the case of four-species co-infections, *E. ninakohlyakimovae* was most common (*n* = 13). Six- species co-infections have not been recorded.

The mean prevalence of infection with nematodes was comparable (χ^2^ = 0.41, *p* = 0.523) in both types of farms, but intensity of infection was significantly higher in conventional farms than organic farms (Z = −5.71; *p* < 0.001) (Table 6). Significantly higher prevalence in conventional farms was observed only in the case of *Nematodirus* spp. (χ^2^ = 8.64, *p* = 0.003) and *Haemonchus* spp. (χ^2^ = 10.32, *p* = 0.001).

Compared to those on the organic farms, the goats on conventional farms were also found to display a significantly higher intensity of infection with *Trichostrongylus* spp. (Z = −4.15; *p* < 0.001), *Oesophagostomum* spp. (Z = −4.04; *p* < 0.001), *Cooperia* spp. (Z = −4.22; *p* < 0.001), *Haemonchus* spp. (Z = −2.48; *p* = 0.013), *Nematodirus* spp. (Z = −2.74; *p* = 0.006).

Conventional farms demonstrated a significantly higher prevalence of infection with *Moniezia expansa* (χ^2^ = 4.52; *p* = 0.033) compared to organic farms. In the case of *Fasciola hepatica*, higher prevalence was observed in conventional farms than in organic farms (6.10% vs. 2.60%), but the difference was not significant (*p* = 0.290).

The occurrence of particular species of helminths in single and multi-species infections in goats on organic and conventional farms are presented in Table 7. Of these, five-species co-infections dominated in organic farms, and four-species co-infections in conventional farms.

## 4. Discussion

The level of infection in goats is influenced by a range of environmental factors that favour the development of the parasitic stages outside the host or limit their survival. On farms, these factors largely depend on the management strategies intended to prevent and control endoparasitic diseases in goat farming systems [26]. Rahmann and Seip [26] reported a greater spread of parasites when goats were kept on deep bedding and were maintained in an alcove-pasture rearing system. In contrast, a lower prevalence of infection was observed among goats maintained in an alcove system, with daily manure removal, as well as during winter, which may be due to the reduced chance of contact between the host and the parasites. The prevalence and intensity of infection were also influenced by the size of the herd, stocking density and choice of prophylaxis programs. Our present findings indicating high prevalence of gastrointestinal parasite infection are consistent with those of previous studies [27,28,29]. 

In goat breeding, a serious problem is presented by parasitosis caused by gastrointestinal nematodes and *Eimeria* protozoa, as well as by flukes such as *Fasciola hepatica* and *Moniezia* tapeworms [30,31,32,33,34,35,36].

Although *Eimeria* spp. generally occur worldwide in goats, no geographical predisposition has been observed for any particular species. Any observed diversity in the prevalence and distribution of coccidiosis is influenced more by the hygiene and temperature in the farm, as well as microclimate, host resistance and susceptibility of the breed to coccidia [37]. Our present findings indicate that *Eimeria* species are widespread in the studied herds. The mean prevalence of *Eimeria* spp. in conventional and organic farms was 85.4% and 77.6%, respectively. These results are similar to those noted in Iran (83.4%) [31], India (79.2%) [32] and in Turkey (73.6%) [38]. A higher prevalence has been reported in Portugal (98.61%) [39], Florida, USA (97%) [40], China (92.9%) [41] and Spain (96.1%) [42]. However, all the cited works concerned research conducted on conventional farms. Unfortunately, only a small number of papers are available on the differences in the prevalence of coccidiosis between organic and conventional animal husbandry methods. However, similar studies have been carried out in herds of cows in West Pomerania (Poland) [6], as in the present studies, the results indicate no significant differences in the prevalence of *Eimeria* spp. between both herd types. In the present study, the presence of a relatively high prevalence of *Eimeria* spp. in both farm types probably results from the contamination of the local habitat with *Eimeria* oocysts. Höglund et al. [43] recommend that gastrointestinal parasites can be controlled by good management, such as the use of parasite-safe pastures. It is possible that the studied farms were not employing enough effective grazing management strategies. Additionally, due to the lack of clinical symptoms in goats, no coccidiostats were used in any of the farms. This could result in a number of oocyst seeders being present in the herd, which would act as a source of infection for healthy animals.

For most *Eimeria* species, no significant difference in prevalence was observed between the organic and conventional farms. The exception was *E. arloingi*, which demonstrated a significantly (*p* = 0.023) higher prevalence in conventional farms than organic farms. This difference is difficult to explain. *E. arloingi* is one of the most pathogenic *Eimeria* species to goats [9]. It may be the case that the animals possessed greater immune resistance or that the studied conventional farms may have had a previous history of coccidiosis. As indicated by Silva et al. [39], protective immune responses against *Eimeria* infections are inhibited, among others, by stress, which can be caused by numerous factors, such as herd size, diet changes, weather conditions or nutritional status. Thus, the lower standard of welfare of goats on conventional farms could have made them more susceptible to infection than those on the organic farms.

In goats, the most pathogenic species of *Eimeria* are believed to be *E. arloingi, E. ninakohlyokimovae*, *E. caprovina*, *E. christenseni*, *E. faurei* and *E. gilruthi* [9,37,44,45,46]. It is worth noting that of these, the most pathogenic species, *viz. E. ninakohlyakimovae*, *E. arloingi* and *E. christenseni*, were quite commonly observed in the studied herds (Table 3). In general, seven species were identified in faecal samples from both organic and conventional farms: *E. arloingi, E. ninakohlyakimovae, E. caprina, E. alijevi, E. jolchijevi, E. hirci and E. chrisienseni*. Değer et al. [38] reported nine different species of *Eimeria* in goats from Turkey, these being *E. arloingi*, *E. christensini*, *E. alijevi*, *E. hirci*, *E. ninakohliyakimovae* and *E. jolchijevi*, in addition to *E. pallida E. apsheronica* and *E. punctata*. Nine species of *Eimeria* were also identified by Kahan and Greiner [40] in goats from Florida, USA. This group included both *E. punctata* and *E. caprovina*. In turn goats from Egypt were found to be infected with *E. ninakohlyakimovae*, *E. hirci*, *E. caprina*, *E. christenseni*, *E. jolchijevi*, *E. apsheronica* and *E. arloingi* [47].

The most prevalent *Eimeria* species on the organic farms were found to be *E. chrisienseni* (40.8%), E. alijevi (40.8%) and *E. arloingi* (36.8%), while the most common on the conventional farms were *E. arloingi* (54.9%*), E. ninakohlyakimovae* (48.8%) and E. *chrisienseni* (41.5%). In previous studies conducted in this area, *E. arloingi*, *E. alijevi*, E. *ninakohliyakimovae* and E. *chrisienseni* were the most common species in goats [48]. Our results are also similar to those of Değer et al. [38]. In turn, Mohamaden et al. [47] noted that the most prevalent species in goats from Egypt were *E. arloingi* (37.04%), *E.ninakohlyakimovae* (30.86%) and *E. hirci* (24.69%).

Epizootiological studies have found mixed coccidial infections played a significant role in goat health around the world [9,31,32,49]. Indeed, in our study, the most common mixed infections were those comprising three and four species of coccidia. Similarly, Değer et al. [38] reported that multiple infections with three (19.4%) or four species (17.4%) were not rare. In addition, Değer et al. [38] also reported the presence of mixed infections in 66.9% of examined goats. This is a lower figure than in the present study, where 73.7% goats from the organic farms demonstrated mixed infection and 81.7% conventional farms.

As reported by Mohamaden et al. [47] 700 oocysts/g indicated a subclinical infection of goats. The number of oocysts ranged from 1000 to 1 × 10^6^ oocysts/g faeces in the faeces of asymptomatic animals and from 100 to 10 × 10^6^ oocysts/g faeces in symptomatic animals [50]. In our study, the mean intensity of infection with *Eimeria* spp. was higher than 1000 opg, but no clinical signs of coccidiosis were observed in either the organic or the conventional farms. This may be due to the fact that adult animals are protected by the cellular immune responses induced by primary *Eimeria* infections [51]. This can lead to the development of enzootic stability between host and parasite and non-clinical status in goats.

A significantly higher intensity of infection with *Eimeria* spp. was observed in goats from conventional farms than those on organic farms (*p* = 0.031). Chartier and Paraud [9] indicated that breeding intensification, high stocking rates, poor hygiene and physiological and nutritional stress all represented risk factors for high excretion. These factors were not observed to a high degree in the present farms. A greater intensity of infection was noted in farms characterised by higher breeding and stocking rates. In addition, Ruiz et al. [42] found oocyst shedding intensity to be related to herd size.

The dominant endoparasites in goats are gastrointestinal nematodes, mainly *Haemonchus contortus, Teladorsagia circumcincta, Trichonstrongylus* spp., all of which also demonstrated high pathogenicity [34]. A range of gastrointestinal nematodes were also identified in the herds in the present study, including *Chabertia ovina*, *Trichostrongylus* spp., *Oesophagostomum* spp., *Cooperia* spp., *Haemonchus* spp., *Nematodirus* spp. and *Strongyloides* spp. (Table 6). In organic farms from the Czech Republic, the most prevalent nematodes were *H. contortus* (42%), *Trichostrongylus* spp. (23%), *Oesophagostomum columbianum* (13%), and *Teladorsagia circumcincta* (11%) [5].

In conventional herds, nematode infections can be controlled by anthelmintic treatments, but this is prohibited, or at least limited, in organic production. However, excessive use of drugs from a single chemical group can result in the parasites developing resistance to the active substances and the consequent failure of treatment [22,52]. In addition, the use of inadequate doses (e.g., for sheep) may also result in the emergence of drug resistance in the nematode population due to its faster breakdown and elimination [53]. This is probably the cause of the significantly (*p* < 0.05) higher prevalence (*Haemonchus* spp., *Nematodisrus* spp.) and intensity of infection (*Trichostrongylus* spp., *Oesophagostomum* spp., *Cooperia* spp., *Haemonchus* spp, *Nematodirus* spp.) of some of the studied nematodes in the goats from conventional farms. The conventional farm owners reported that goats were treated with levamisole twice a year without prior testing. In contrast, the goats on organic farms were only treated in case of severe infection. The available literature indicates that levamisole has low efficacy against nematode in goats, ranging from 43.4 to 52.6% in the period 10–60 days after administration [52]. Generally, this could be one of the reasons for the high prevalence of gastrointestinal nematodes infections, which was above 80% in both types of farms. This prevalence was higher than that reported by Dey et al. [54] in Bangladesh (62.1%), Zvinorova et al. [55] (31.0–40.0%) in Zimbabwe and Jegede et al. [56] in Nigeria (37.5%). In contrast, a very low prevalence of nematodes has been reported in organic goat herds in Greece (7.4%) [57]. Authors [57] reported the presence of strongyle-type eggs in 3.4% samples, *Nematodirus* spp. eggs in 1.1% samples and *Trichuris* spp. eggs in 2.9% samples.

In the present study, the dominant nematode was found to be *Haemonchus* spp. in the conventional farms (59.8%) and *Trichostrongylus* spp. in the organic farms (56.6%). While no significant differences in prevalence were observed between the two types of farms, in the case of *Trichostrongylus* spp., significant differences (*p* = 0.001) were observed for *Haemonchus* spp. This situation can be attributed to some goat breeds possessing a genetic resistance to nematode infections. Comparative studies found that Boer goats, the breed kept on organic farms, demonstrated the highest expression of the DRB1∗1101 gene after exposure to *Haemonchus contortus* [58].

Animals are typically infected with several species of nematodes at the same time. The prevalence of mixed infection in this study was 73.7% on organic forms and 81.7% on conventional ones. Elsewhere, 46% of goats were found to have mixed infections in Argentina [59], while only 6.25% of goats from Ethiopia demonstrated mixed infection by gastrointestinal nematodes [60].

In organic farms, much attention was paid to grazing management [27], the purpose of which is to prevent and control endoparasite diseases. The farms included in the present study employed strategies based on shortening the grazing period and sowing plants with antiparasitic properties: A number of plants, such as garlic (*Allium sativum*), mugwort (*Artemisia absinthium*), black walnut (*Juglans nigra*), mugwort (*Artemisia vulgaris*) and common thyme (*Thymus vulgaris*), are known to demonstrate such properties [61,62,63]. In addition, a restricted living environment, such as a farm, is associated with a higher risk of parasitic infections [64]. Therefore, it is possible that the goats kept on organic farms demonstrated a lower intensity of infection with gastrointestinal nematodes due to the fact that lower numbers of animals were kept in a given area and that they may have been grazing on plants exhibiting antiparasitic properties. In addition, Saanen goats were kept on the organic farm. This breed is known to carry three single-nucleotide polymorphisms (SNPs) pertaining to four candidate genes of the cytokine family (IL2, IL4, IL13, and IFNG), which may be associated with its greater resistance to gastrointestinal endoparasitic infections [58].

In the present study, the only tapeworm found on both the organic and conventional farms was *Moniezia expansa*. Similar results in goats were obtained by Górski et al. [35]. However, they report a significantly lower prevalence (2–2.6%) compared to our present findings (17.1% and 31.7%, Table 6). It is possible that the higher prevalence of *Moniezia* spp. observed in the present study was due to the samples being collected in autumn (September): the prevalence of *Moniezia* spp. is conditioned by the periods of activity of oribatid mites, their intermediate hosts, which is greater in summer or autumn [65,66].

*Moniezia* spp. was noted in 31% of goats from Nigeria [67], 12% in Ethiopia [68], and 2.64% in Slovakia [69]. Fagbemi and Dipeolu [70] reported that the prevalence of *Moniezia* spp. was generally low in animals kept on an extensive system of management with low stocking rates. This could also explain the significantly (*p* = 0.033) lower prevalence of this tapeworm in goats from organic farms.

In the present study, significant differences in the prevalence of *Moniezia* spp. were found between the two types of farms, but both demonstrated similar intensity of infection (150 and 165 EPG). A significantly higher intensity of infection was noted in Slovakia (320 EPG) [69].

Fagbemi and Dipeolu [70] note that low pathogenicity of *Moniezia* spp. was usually associated with low-grade infections. However, this pathogenicity can increase if an animal is coinfected with other parasites. For example, mixed infection of *Moniezia* and *Trichuris* can result in severe malnutrition, leading to pulmonary oedema and the death of the animal [71]. In the organic farms, *Moniezia* spp. was present in mixed infections of three to eight species, while in the conventional farms, it was observed as both single infections and mixed infections containing from two to eight species of the parasite (Table 7). However, no clinical signs were observed in animals, possibly due to the low intensity of infection.

The liver fluke *Fasciola hepatica*, the causative agent of fasciolosis, is associated with wetlands or periodically flooded areas. Such areas are a habitat for intermediate hosts such as the snail *Galba truncatula.* The course and symptoms of the disease depend on the age of the hosts, their nutritional status and the intensity of infection [72,73]. Our findings indicate that the occurrence of *F. hepatica* in goats is rare in Poland, as also indicated by Górski et al. [35]. This fluke was also found sporadically in Greece. According to Kantzoura et al. [57], the prevalence was 0.5% in sheep from organic farms and from 0 to 2.5% in those from conventional farms, depending on the region.

Fluke infection is usually diagnosed by coproscopic examination based on decantation, mainly due to its simplicity. However, this method is only effective three to four months after infection, when sexually mature flukes start producing eggs. In addition, as egg production by flukes is not constant, the method has relatively low sensitivity [74]. As a result, the true prevalence of *F. hepatica* may be underestimated. Even so, it must be remembered that the appearance of liver flukes in a herd is a significant problem for farmers. Fluke infection may increase the susceptibility to infection with other parasites. For example, Cuervo et al. [59] noted significant (*p* < 0.05) positive associations between *F. hepatica* and Strongyle eggs. The authors suggest that infection by *F. hepatica* may act as a contributing factor for Strongyle infection, increasing the chance almost twofold (1.96 and 1.83, respectively), probably due to the host immunosuppression caused by the trematode, or possibly due to the weakened state of the host.

In this study, no significant differences in prevalence of *F. hepatica* were observed between two types of farms. However, this may be due to the small number of infected animals: two in the organic farms and five in the conventional farms. In contrast, a higher prevalence of *F. hepatica* was observed in cows from organic farms than those from conventional farms in Denmark [75]. However, the authors [75] note that the conventional farms implemented *F. hepatica* control strategies based on grazing management, resulting in a decreased level of infection. Olsen et al. [76] suggest that the higher prevalence of *F. hepatica* in organic animals may arise from greater access to pasture or to lower treatment levels in organic herds.

## 5. Conclusions

In general, our findings indicate that goats in conventional herds demonstrate a higher prevalence of parasitic diseases than organic herds. Only *E. arloingi*, *Haemonchus* spp. *Nematodirus* spp., *Moniezia* spp. and *Fasciola hepatica* demonstrated a greater prevalence in conventional farms. The prophylactic programs used to combat parasitic infections in both types of farms appear ineffective and require improvement. There is a need for goat herds to be covered by ongoing parasitological monitoring, including parasitological testing before and after the pasture season, to detect carriers and shedders of parasite eggs. It is also recommended that keepers employ rotational or intensive rotational grazing methods and take care to ensure the hygiene of animal quarters and livestock rooms. Furthermore, accurate diagnosis of parasitic infections, as well as effective monitoring and prophylaxis, are essential for keeping goat herds free from parasites.

## Figures and Tables

**Table 1:** Characteristic of organic and conventional farms.

	Organic Farms	Conventional Farms
Breed	Saanen goat Boer goat	Polish White Improved goat
Age (years)	• 3–7	
Nutrition	Pasture from April to October/November, (weather dependent), hay, crushed oats. Salt licks from Kłodawa salt with Se. Winter: barley straw, dried legumes	Pasture from April to October/November, (weather dependent), hay, crushed oats. Salt licks from Kłodawa salt with Se. Winter: barley straw, dried legumes
Housing and microclimate	• Deep bedding • Within the normal range	
Reproduction	• Natural mating	
Hoof trimming	• If needed, mainly 2 times/year (in spring before pasture, and in autumn after pasture)	
Deworming	If needed, after examination	2 times/year (in spring before pasture, and in autumn after pasture)
Disinfection of animal housing	• 1 time a year—after removing the manure	
Soil	Chernozem and rendzinas	Podzolic, brown soils, rendzinas

**Table 2:** Mean prevalence of infection (E.I%) among goats on the tested farms.

	Conventional Farms	Organic Farms
Number of tested animals	82	76
Number of infected animals	72	64
Prevalence (%)	87.80	84.21

**Table 3:** Prevalence and intensity of infection with *Eimeria* spp. among the tested goats.

Parasite	Type of Farm	Number of Goats Infected/Tested	Prevalence (%) (95%CI)	χ^2^ Test Value		Intensity of Infection		
					Mean	Median	Range	Mann–Whitney U-test Value
*Eimeria* spp.								
*E. arloingi*	O	28/76	36.8 (26.9–48.1)	χ^2^ = 5.16 *p* = 0.023	755.4	550	50–3500	Z = −0.59 *p* = 0.554
	C	45/82	54.9 (44.1–65.2)		1024.4	600	50–9500	
*E. ninakohlyakimovae*	O	26/76	34.2 (24.5–45.4)	χ ^2^ = 3.44 *p* = 0.063	432.7	400	50–1300	Z = −0.13 *p* =0.900
	C	40/82	48.8 (38.3–59.4)		453.8	350	50–1450	
*E. caprina*	O	29/76	38.2 (28.0–49.4)	χ^2^ = 0.13 *p* = 0.716	169.0	150	50–450	Z = −0.12 *p* = 0.905
	C	29/82	35.4 (25.9–46.2)		272.4	100	50–1500	
*E. alijevi*	O	31/76	40.8 (30.4–52.0)	χ^2^ = 1.83 *p* = 0.176	456.5	150	50–4000	Z = 0.42 *p* = 0.676
	C	25/82	30.5 (21.6–41.2)		306.0	100	50–1300	
*E. jolchijevi*	O	13/76	17.1 (10.1–27.2)	χ^2^ = 1.27 *p* = 0.260	115.4	50	50–600	Z = −2.88 *p* = 0.004
	C	20/82	24.4 (16.3–34.8)		317.5	150	50–1300	
*E. hirci*	O	19/76	25.0 (16.6–35.9)	χ^2^ = 1.50 *p* = 0.221	236.8	300	50–450	Z = 0.51 *p* = 0.613
	C	14/82	17.1 (10.3–26.8)		282.1	150	50–1500	
*E. chrisienseni*	O	31/76	40.8 (30.4–52.0)	χ^2^ = 0.01 *p* = 0.931	454.8	300	50–2000	Z = −1.64 *p* = 0.101
	C	34/82	41.5 (31.4–52.3)		1088.2	550	50–4500	
Total *Eimeria* spp.	O	59/76	77.6 (67.0–85.6)	χ ^2^ = 1.57 *p* = 0.209	1204.3	950	50–4400	Z = −2.15 *p* = 0.031
	C	70/82	85.4 (76.0–91.6)		1815.7	1400	50–9850	

O—organic farms; C—conventional farms.

**Table 4:** Occurrence of particular species of *Eimeria* spp. in single and multi-species infections in goats on organic farms.

Parasite	Number of Infected Goats	Form of Infections Number of Goats (n), Prevalence (%)					
		1-Species	2-Species	3-Species	4-Species	5-Species	6-Species
*E. arloingi*	28	5 (17.86)	3 (10.71)	6 (21.43)	8 (28.57)	2 (7.14)	4 (14.29)
*E. ninakohlyakimovae*	26	1 (3.85)	5 (19.23)	5 (19.23)	7 (26.92)	2 (7.69)	6 (23.08)
*E. caprina*	29	1 (3.45)	4 (13.79)	8 (27.59)	6 (20.69)	4 (13.79)	6 (20.69)
*E. alijevi*	31	1 (3.23)	3 (9.68)	4 (12.90)	11 (35.48)	6 (19.35)	6 (19.35)
*E. jolchijevi*	13	2 (15.38)	2 (15.38)	2 (15.38)	1 (7.69)	2 (15.38)	4 (30.77)
*E. hirci*	19	0	1 (5.26)	2 (10.53)	10 (52.63)	2 (10.53)	4 (21.05)
*E. chrisienseni*	31	3 (9.68)	5 (16.13)	8 (25.81)	6 (19.35)	4 (12.90)	5 (16.13)
Total	--	13 (7.34)	23 (12.99)	35 (19.77)	49 (27.68)	22 (12.43)	35 (19.77)

**Table 5:** Occurrence of particular species of *Eimeria* protozoans in single and multi-species infections in goats on conventional farms.

Parasite	Number of Infected Goats	Form of Infections Number of Goats (*n*), Prevalence (%)						
		1-Species	2-Species	3-Species	4-Species	5-Species	6-Species	7-Species
*E. arloingi*	45	3 (6.67)	6 (13.33)	18 (40.00)	11 (24.44)	6 (13.33)	0	1 (2.22)
*E. ninakohlyakimovae*	40	1 (2.50)	4 (10.00)	16 (40.00)	13 (32.5)	5 (12.5)	0	1 (2.5)
*E. caprina*	29	2 (6.90)	4 (13.79)	7 (24.14)	11 (37.93)	4 (13.79)	0	1 (3.45)
*E. alijevi*	25	2 (8.00)	1 (4.00)	7 (28.00)	11 (44.00)	3 (12.00)	0	1 (4.00)
*E. jolchijevi*	13	0	3 (23.08)	2 (15.38)	5 (38.46)	2 (15.38)	0	1 (7.69)
*E. hirci*	19	0	7 (36.84)	3 (15.79)	3 (15.79)	5 (26.32)	0	1 (5.26)
*E. chrisienseni*	34	3 (8.82)	9 (26.47)	7 (20.59)	7 (20.59)	7 (20.59)	0	1 (2.94)
Total	--	11 (5.37)	34 (16.59)	60 (29.27)	61 (29.76)	32 (15.61)	0 (0.00)	7 (3.41)

**Table 6:** Prevalence and intensity of infections with helminths in the studied goats.

Parasite	Type of Farm	Number of Goats Infected/Tested	Prevalence (%)(95% CI)	χ^2^ Test Value		Intensity of Infection		
					Mean	Median	Range	Mann–Whitney U-Test Value
Gastrointestinal Nematodes								
*Chabertia ovina*	O	37/76	48.7 (37.8–59.7)	χ^2^ = 0.09 *p* = 0.768	150.0	100	50–600	Z = −1.52 *p* = 0.129
	C	38/82	46.3 (36.0–57.1)		552.6	125	50–5000	
*Trichostrongylus* spp.	O	43/76	56.6 (45.4–67.1)	χ^2^ = 1.28 *p* = 0.257	120.9	50	50–600	Z = −4.15 *p* < 0.001
	C	39/82	47.6 (37.1–58.2)		350.0	300	50–1550	
*Oesophagostomum* spp.	O	29/76	38.2 (28.0–49.4)	χ^2^ = 0.07 *p* = 0.788	160.3	50	50–600	Z = −4.04 *p* < 0.001
	C	33/82	40.2 (30.3–51.1)		516.7	350	50–3000	
*Cooperia* spp.	O	22/76	29.0 (19.9–40.0)	χ^2^ = 1.78 *p* = 0.182	90.9	50	50–300	Z = −4.22 *p* < 0.001
	C	32/82	39.0 (29.2–49.9)		404.7	300	50–1500	
*Haemonchus* spp.	O	26/76	34.2 (24.5–45.4)	χ^2^ = 10.32 *p* = 0.001	134.6	100	50–500	Z = −2.48 *p* = 0.013
	C	49/82	59.8 (48.9–69.7)		317.3	150	50–1000	
*Nematodirus* spp.	O	19/76	25.0 (16.6–35.9)	χ^2^ = 8.64 *p* = 0.003	105.3	50	50–600	Z = −2.74 *p* = 0.006
	C	39/82	47.6 (37.1–58.2)		306.4	100	50–2000	
*Strongyloides* spp.	O	35/76	46.1 (35.3–57.2)	χ^2^ = 0.54 *p* = 0.461	270.0	300	50–600	Z = 0.32 *p* = 0.752
	C	33/82	40.2 (30.3–51.1)		328.8	150	50–1200	
Total	O	61/76	80.3 (69.8–87.8)	χ^2^ = 0.41 *p* = 0.523	519.3	500	50–1450	Z = −5.71 *p* < 0.001
	C	69/82	84.2 (74.6–90.6)		1492.8	1200	100–7900	
Tapeworm								
*Moniezia expansa*	O	13/76	17.1 (10.1–27.2)	χ^2^ = 4.52 *p* = 0.033	150.0	100	50–350	Z = −0.15 *p* = 0.878
	C	26/82	31.7 (22.6–42.4)		165.4	100	50–600	
Fluke								
*Fasciola hepatica*	O	2/76	2.63 (0.02–9.6)	χ^2^ = 1.12 *p* = 0.290	50	50	50–50	--
	C	5/82	6.10 (2.3–13.8)		140	108.4	50–300	

O—organic farms; C—conventional farm; --—no data.

**Table 7:** Occurrence of particular species of helminths in single and multi-species infections in goats on organic and conventional farms.

Parasite	Number of Infected Goats	Form of Infections-Number of Goats (Prevalence%)							
		1-Species	2-Species	3-Pecies	4-Species	5-Species	6-Species	7-Species	8-Species
Organic farms									
*Chabertia ovina*	40	3 (7.50)	2 (5.00)	7 (17.50)	6 (15.00)	12 (30.00)	8 (20.00)	0	2 (5.00)
*Trichostrongylus* spp.	45	2 (4.44)	3 (6.67)	10 (22.22)	7 (15.56)	14 (31.11)	7 (15.56)	0	2 (4.44)
*Oesophagostomum* spp.	31	0	1 (3.23)	7 (22.58)	10 (32.26)	6 (19.35)	5 (16.13)	0	2 (6.45)
*Cooperia* spp.	24	0	3 (12.50)	3 (12.50)	729.17)	5 (20.83)	4 (16.67)	0	2 (8.33)
*Haemonchus* spp.	27	1 (3.70)	3 (11.11)	2 (7.41)	4 (14.81)	10 (37.04)	5 (18.52)	0	2 (7.41)
*Nematodirus* spp.	19	0	2 (10.53)	1 (5.26)	1 (5.26)	7 (36.84)	6 (31.58)	0	2 (10.53)
*Strongyloides* spp.	34	2 (5.88)	2 (5.88)	6 (17.65)	5 (14.71)	12 (35.29)	5 (14.71)	0	2 (5.88)
*Moniezia expansa*	12	0	0	3 (25.00)	1 (8.33)	5 (41.67)	1 (8.33)	1 (8.33)	1 (8.33)
*Fasciola hepatica*	2	0	0	1 (50.00)	0	0	1 (50.00)	0	0
total	--	8 (5.97)	16 (11.94)	40 (29.85)	41 (30.60)	71 (92.99)	42 (31.34)	1 (0.75)	15 (11.19)
Conventional farms									
*Chabertia ovina*	34	0	2 (5.88)	4 (11.76)	8 (23.53)	9 (26.47)	6 (17.65)	5 (14.71)	0
*Trichostrongylus* spp.	37	2 (5.41)	2 (5.41)	1 (2.70)	15 (40.54)	7 (18.92)	5 (13.51)	5 (13.51)	0
*Oesophagostomum* spp.	33	0	3 (9.09)	4 (12.12)	8 (24.24)	9 (27.27)	4 (12.12)	5 (15.15)	0
*Cooperia* spp.	30	0	4 (13.33)	3 (10.00)	14 (46.67)	3 (10.00)	2 (6.67)	4 (13.33)	0
*Haemonchus* spp.	48	0	1 (2.08)	5 (10.42)	17 (35.42)	12 (25.00)	8 (16.67)	5 (10.42)	0
*Nematodirus* spp.	39	0	3 (7.69)	2 (5.13)	17 (43.59)	5 (12.82)	6 (15.38)	6 (15.38)	0
*Strongyloides* spp.	32	0	1 (3.13)	2 (6.25)	8 (25.00)	8 (25.00)	7 (21.88)	6 (18.75)	0
*Moniezia expansa*	27	3 (11.11)	2 (7.41)	3 (11.11)	6 (22.22)	6 (22.00)	4 (14.81)	3 (11.11)	0
*Fasciola hepatica*	4	0	0	0	2 (50.00)	1 (25.00)	1 (25.00)	0	0
Total	--	5 (1.76)	18 (6.34)	24 (8.45)	95 (33.45)	60 (21.13)	43 (15.14)	39 (13.73)	0

## Data Availability

The data presented in this study are available on request from the corresponding author.
